# A new phenological metric for use in pheno‐climatic models: A case study using herbarium specimens of *Streptanthus tortuosus*

**DOI:** 10.1002/aps3.11276

**Published:** 2019-07-12

**Authors:** Natalie L. Rossington Love, Isaac W. Park, Susan J. Mazer

**Affiliations:** ^1^ Department of Ecology, Evolution, and Marine Biology University of California, Santa Barbara Santa Barbara California 93106 USA

**Keywords:** climate change, herbarium, herbarium specimens, pheno‐climatic models, phenological index, phenology

## Abstract

**Premise:**

Herbarium specimens have been used to detect climate‐induced shifts in flowering time by using the day of year of collection (DOY) as a proxy for first or peak flowering date. Variation among herbarium sheets in their phenological status, however, undermines the assumption that DOY accurately represents any particular phenophase. Ignoring this variation can reduce the explanatory power of pheno‐climatic models (PCMs) designed to predict the effects of climate on flowering date.

**Methods:**

Here we present a protocol for the phenological scoring of imaged herbarium specimens using an ImageJ plugin, and we introduce a quantitative metric of a specimen's phenological status, the phenological index (PI), which we use in PCMs to control for phenological variation among specimens of *Streptanthus tortuosus* (Brassicaceeae) when testing for the effects of climate on DOY. We demonstrate that including PI as an independent variable improves model fit.

**Results:**

Including PI in PCMs increased the model *R* ^2^ relative to PCMs that excluded PI; regression coefficients for climatic parameters, however, remained constant.

**Discussion:**

Our protocol provides a simple, quantitative phenological metric for any observed plant. Including PI in PCMs increases *R* ^2^ and enables predictions of the DOY of any phenophase under any specified climatic conditions.

Studies of phenology—the timing of life cycle events—have provided some of the strongest evidence that many organisms have been or will be affected by global changes in climate (Parmesan and Yohe, 2003; Menzel et al., 2006). Plants are sensitive to changes in climate, especially changes in temperature, and plant phenology has been monitored and tracked through time using a variety of approaches, including long‐term in situ observations of living plants (Sparks and Carey, 1995; Chmielewski and Rötzer, 2001; Rutishauser et al., 2009), citizen science networks (Mayer, 2010; Haggerty et al., 2013), satellite imagery (Stöckli and Vidale, 2004; Studer et al., 2007; White et al., 2009), and herbarium specimens (Lavoie and Lachance, 2006; Panchen et al., 2012; Hufft et al., 2018).

Because of their long temporal record and expansive geographic range, herbarium specimens have been used to detect species‐specific shifts in phenology through time in response to changing climate (Lavoie, 2013; Willis et al., 2017; Jones and Daehler, 2018). Herbarium‐based studies have detected temporal advancement in phenology and have quantified the sensitivity of phenology to climatic parameters such as temperature and precipitation. Given the value of herbarium specimens in studying the effects of climate change on the seasonal cycles of plants, several recent collaborative efforts have aimed to digitize and to provide electronic access to the images and label information of millions of herbarium specimens currently housed in separate herbaria (Willis et al., 2017; Yost et al., 2018). If these efforts are successful, then herbarium specimens will be widely available for study and provide a wealth of easily accessible new data with which to investigate phenological patterns over space and time.

Herbarium‐based studies designed to link phenology to local climatic conditions typically rely on the day of year of collection (DOY) of specimens that were collected in flower. In these studies, DOY is considered to be a proxy for first flowering date (FFD) or the date of peak flower (DPF) (Primack et al., 2004; Diskin et al., 2012; Davis et al., 2015), two phenological events that are commonly used to track phenology in field‐based observations. The DOY is then used as a dependent variable and regressed against either the year of collection or one or more climate parameters during the year of specimen collection (or during the months preceding it) in order to detect temporal shifts in phenology or to quantify the sensitivity of plants to specific climatic parameters.

Using DOY as a proxy for flowering time is problematic because reproductive herbarium specimens may have been collected at any point between bud formation and fruit ripening; therefore, the DOY may not accurately represent either FFD or DPF. We can use a hypothetical regression to visualize two inaccuracies that may occur by using the DOY of reproductive specimens as a proxy for either of these phenological metrics (Fig. 1). First, the DOY of a flowering specimen will always and necessarily be on or after its true FFD (Fig. 1A). Second, the DOY may be before, after, or on the true DPF (Fig. 1B–D). Specimens may be preferentially collected before the true DPF if the floral structures are fragile or ephemeral, and may be preferentially collected after the true DPF if fruits are necessary for correct plant identification or are particularly showy (Fig. 1B and 1C, respectively). If specimens are collected evenly throughout their reproductive period, then DOY may accurately predict the true DPF (Fig. 1D).

**Figure 1:**
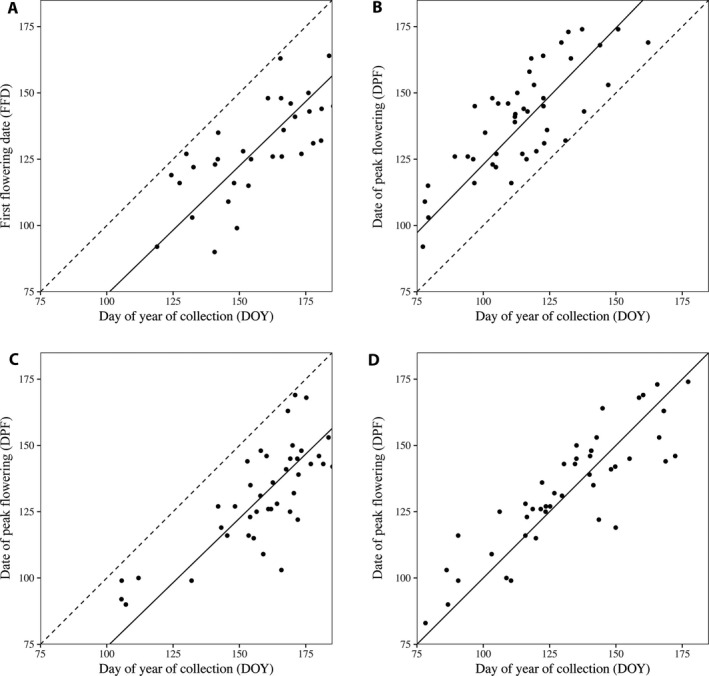
The hypothetical relationships between day of year of collection (DOY;* x*‐axis) and (A) first flowering date (FFD) and (B–D) day of peak flowering (DPF). The dotted line shows a 1 : 1 relationship, where DOY is a perfect proxy for the dependent variable. Figure 1A shows that the FFD is necessarily earlier than the DOY (there are no values of DOY that are above the line representing the 1 : 1 relationship between FFD and DOY). Figure 1B–D show the three hypothetical relationships between DPF and DOY where DOY may be (B) before, (C) after, or (D) on the true DPF but is rarely an accurate representation of the true DPF.

Figure 1 demonstrates a case in which DOY and FFD or DPF are strongly positively correlated among specimens, but DOY does not accurately predict either FFD or DPF because specimens may not be collected on their true FFD or DPF. If this situation is common, then assuming that DOY accurately represents FFD or DPF when investigating relationships between phenology and climate would reduce the explanatory power of the resulting models because of the high variation in phenological stage among herbarium sheets, and the fact that variation in DOY caused by variation in the actual phenophase of collection (i.e., FFD or DPF) is conflated with variation in the timing of collection of a given specimen relative to the actual timing of FFD or DPF. This effect is likely to be particularly intense among species that exhibit long flowering durations, as longer flowering durations increase the maximum potential difference in the timing of collection DOY from the day of year of actual FFD or DPF. We can see an example of how variation among sheets impacts these analyses by looking at studies that investigate relationships between phenology and climate using both the estimated peak flowering date from herbarium specimens and the true peak flowering date from field observations. Robbirt et al. (2011) compared sensitivities of *Ophrys* orchids using both herbarium specimens and field data. They recorded the DOY of collection of herbarium specimens that were assumed to be in peak flower (excluding those specimens for which fewer than 60% of flowers were open) but likely included specimens that were collected both pre– and post–peak flowering. Data recorded from field‐based observations, by contrast, represented the true dates of peak flower. When the flowering date derived from each data set was regressed (separately) on temperature, both data sets showed a negative relationship between flowering date and temperature, but temperature explained four times more variation in flowering date in the field data–based model than the herbarium data–based model (58.6% vs. 13.4%, respectively), presumably because it did not conflate variation in actual DPF with variation caused by sample collection that occurred before or after DPF. High variation among the phenological status of herbarium sheets is one potential reason for the low explanatory power of models constructed with herbarium‐derived data. Another potential factor includes the possibility that herbarium‐derived data, which are often distributed across broader spatial extents than field‐based data, may therefore also differ from many field‐based data with respect to the range of climatic conditions represented.

Reducing—or controlling statistically for—variation among herbarium or living specimens in their phenological status could help to improve models and to clarify relationships between climate and flowering or collection date. This could be done by either (1) restricting data sets to include only those specimens collected at a specific phenological stage or (2) incorporating into statistical models a quantitative metric that estimates the phenological status of individual specimens or plants. Given that herbarium specimens are rarely collected precisely at first flower or at peak flowering, the first approach would drastically reduce the sample size used to estimate relationships between phenology and climate. This reduction in sample size might preclude the analysis of species represented by relatively few specimens (e.g., <100 sheets). In addition, because herbarium specimens represent an instantaneous snapshot of an individual's phenological status, it is nearly impossible to determine whether an individual specimen was collected at peak flower. However, we can easily quantify a specimen's phenological status by determining the numbers of the different classes of reproductive organs (e.g., buds, flowers, fruits) present on each sheet, and then converting those counts into a proportional weighted mean.

Here, our objectives are (1) to present a protocol designed to score and to record the numbers of reproductive structures representing successive developmental stages on imaged herbarium specimens using a plugin (Cell Counter) developed for the image analysis software ImageJ; (2) to introduce a new integrated metric of a specimen's phenological status—the phenological index (PI)—which is calculated using the counts derived from Cell Counter and allows us to control for the variation in the phenological status of collected specimens when testing statistical models for the effect of climatic conditions on the DOY of specimen collection; (3) to demonstrate how the PI can be used to construct and improve pheno‐climatic models using a herbarium‐derived data set composed of mountain jewelflower (*Streptanthus tortuosus* Kellogg, Brassicaceae) specimens; and (4) to discuss how parameterized models that include the PI as an independent variable can be used as a predictive model and as a means to quantify the length of the reproductive period.

In addition to demonstrating the usefulness of incorporating the PI into pheno‐climatic models, we tested the following three predictions with herbarium‐derived data for *S. tortuosus*. First, given that many studies of plant phenology report that an increase in local winter or spring temperatures (over time or space) induces individual plants or populations to flower earlier (Parmesan and Yohe, 2003; Menzel et al., 2006; Cleland et al., 2007), we predict that, across the localities from which herbarium specimens have been collected, elevated spring temperatures will be associated with earlier flowering in *S. tortuosus*. The relationship between flowering date and precipitation remains unclear and likely differs among species and communities (Hart et al., 2014; Munson and Sher, 2015; Rawal et al., 2015; Matthews and Mazer, 2016). Because the majority of the *S. tortuosus* records analyzed here were collected from localities that experience a Mediterranean climate, their growth or reproduction in the spring and summer may be strongly influenced by winter water availability. Where winter precipitation is relatively low, soils dry out more quickly during the following spring, and this may select for earlier flowering genotypes or induce earlier flowering as a plastic response (Franks, 2011; Hamann et al., 2018). Consequently, our second prediction is that flowering date will be positively correlated with total winter precipitation. Third, as differences in PI among herbarium specimens will account for a portion of the variation in the DOY, we predict that, for data sets comprising specimens among which there is wide variation in the PI, including the PI as an independent variable will result in a model with a higher predictive power than models that do not include PI.

## METHODS

### The phenological index

The PI is an integrative metric derived from the proportions of each class of reproductive units (in this case buds, flowers, immature fruits, and mature fruits) present on a preserved plant on a herbarium sheet. The proportion of a given class is then weighted by an index representing the degree of phenological advancement of that class (e.g., buds = 1; open flowers = 2; immature fruits = 3; and mature fruits = 4). The following equation can be used to calculate the PI for each plant:

(1) $$\sum_{i=1}^{4}\left({\text{p}}_{x}\right)\left(\text{i}\right)=\;\text{phenological index}\;\;\text{(PI)}$$

where p_x_ is the proportion of reproductive units in phenophase x and i is the index assigned to reproductive unit x. The value of PI therefore represents a weighted mean of all of a specimen's reproductive units, where lower values are associated with early development and higher values are associated with more advanced development. For example, if a plant has 50 buds, 40 open flowers, 10 immature fruits, and zero mature fruits, the specimen would have a PI of 1.6, indicating that it is fairly early in its phenological progression.

### Scoring specimens

**Figure 2:**
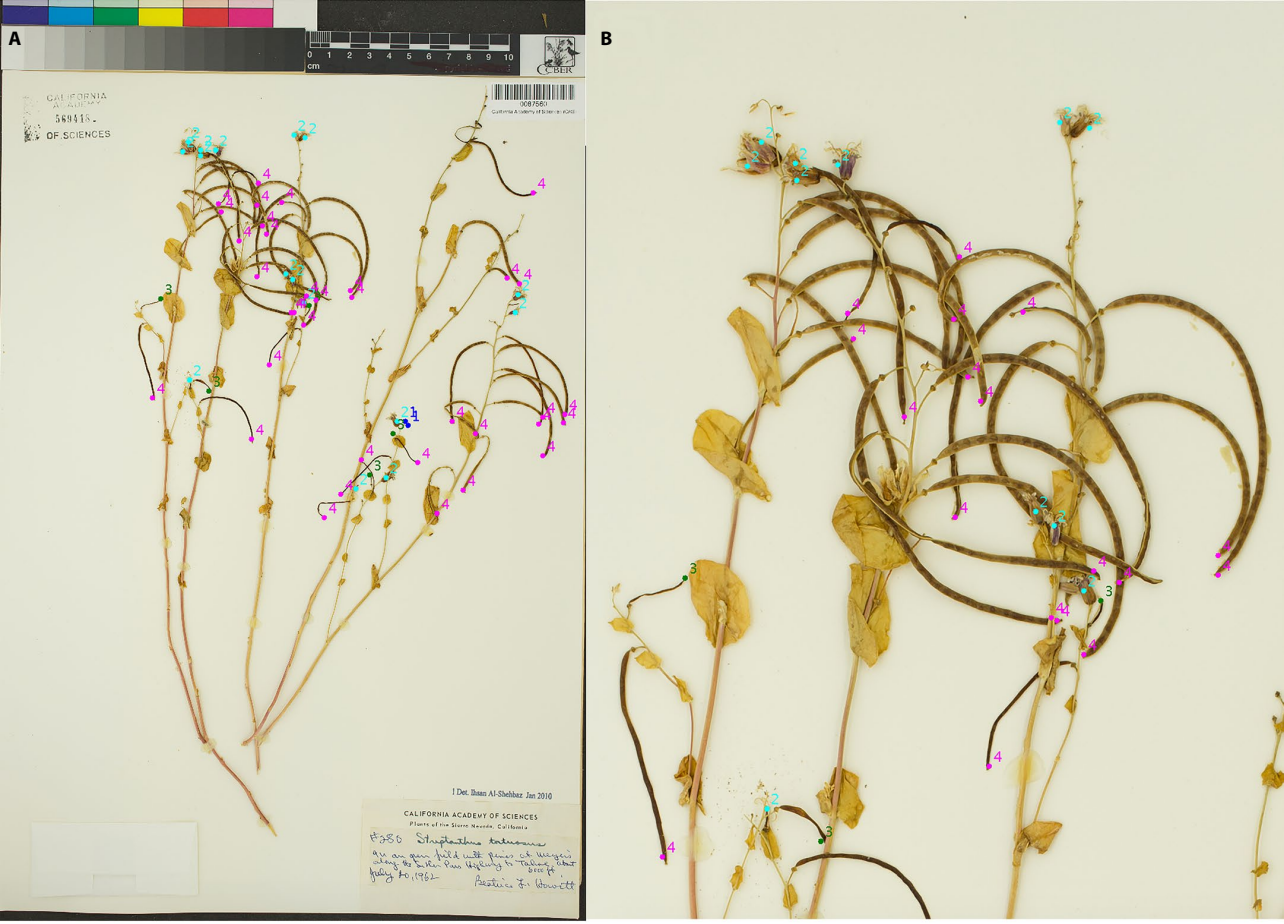
An example of a specimen scored with the ImageJ plugin Cell Counter (CAS0087560) showing (A) the entire herbarium record sheet and (B) a close‐up of a group of scored branches. Each reproductive unit is labeled as a bud (1), flower (2), immature fruit (3), or mature fruit (4). This specimen (which is assumed to represent one plant) has 0 buds, 12 flowers, seven immature fruits, and 26 mature fruits. It has an integrated phenological index of 3.31, which indicates a relatively late stage of phenological progression. The *x‐y* coordinates of all of the individual markers can be saved as an XML text file, which can then be recalled or edited.

One hundred twenty *S. tortuosus* (Brassicaceae) herbarium specimens from the California Academy of Sciences (CAS) and the University of California, Santa Barbara (UCSB), were imaged using an ORTECH Photo e‐Box Plus 1419 imaging station (ORTECH Professional Lighting, Chula Vista, California, USA) at the Cheadle Center for Biodiversity and Ecological Restoration at UCSB. Each plant on the imaged sheets was scored with ImageJ using the plugin Cell Counter by counting the number of buds, flowers, immature fruits, and mature fruits present on each plant (ImageJ version 1.52a available at https://imagej.nih.gov [Abramoff et al., 2004]; Cell Counter plugin available at https://imagej.nih.gov/ij/plugins/cell-counter.html; Fig. 2). Cell Counter, originally developed for counting cells on microscope images, is a simple, fast, and reliable way to score imaged specimens. To score each specimen, the user places digital colored markers that correspond to each reproductive structure and then the program sums the total number of markers in each category, thereby providing an accurate count of the number of buds, flowers, immature fruits, and mature fruits present on each plant (Fig. 2). Cell Counter also allows the user to save the *X‐Y* coordinates of each marker in an XML file that can later be recalled or edited. The protocol we developed and used to score *S. tortuosus* is provided in Appendix 1.

The 120 *S. tortuosus* specimens were scored using Cell Counter according to definitions for each reproductive unit specific to this species (Table 1). One specimen sheet did not have any reproductive plants, and consequently our final data set contained 119 specimens. The counts obtained from Cell Counter for *S. tortuosus* specimens were converted into a phenological index for each plant using Equation 1. For herbarium specimens with more than one plant present on the sheet, the phenological index was averaged across all plants.

**Table 1:** The definitions for buds, flowers, immature fruits, and mature fruits used for scoring *Streptanthus tortuosus* specimens.

Reproductive unit	Definition
Bud	Unopened flower with no petals visible. Must be >2 mm in length to be counted.
Flower	Petal tips visible and/or anthers visible, with the filaments still attached to the receptacle
Immature fruit	Immature ovary with no perianth parts or filaments attached to the receptacle. Contains seeds that are not yet mature.
Mature fruit	Silique with mature seeds and an arc shape. Maturity can be determined if fruit has any evidence of dehiscence or if swollen, mature seeds cause a wavy silique margin.

### Climatic data

**Figure 3:**
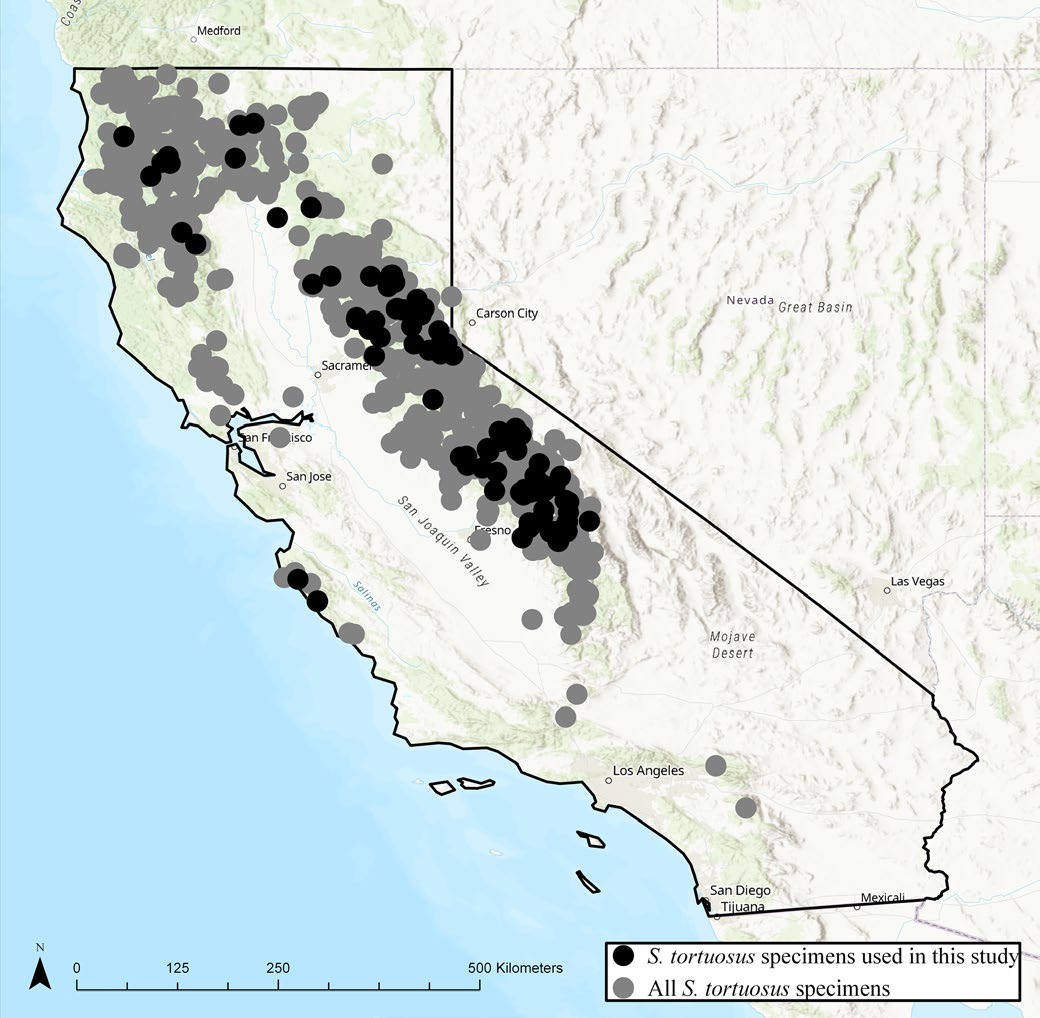
Locations of the collection sites of the *Streptanthus tortuosus* herbarium specimens used in this investigation (black points; *n* = 119) and of all georeferenced *S. tortuosus* specimens currently available in the California Consortium of Herbaria (CCH) database (gray points, *n* = 1719). Our sample represents the geographic and climatic range of *S. tortuosus* well.

Each herbarium specimen was georeferenced by downloading the coordinates and the error radius from the California Consortium of Herbaria (CCH, http://ucjeps.berkeley.edu/consortium/), which is a database that contains location information for many California herbarium records (Fig. 3). These coordinates are georeferenced based on the description of the location on the specimen label. These coordinates were then used to download site‐specific climatic data from PRISM (available at http://prism.oregonstate.edu) during the year and previous year that each herbarium specimen was collected. Specifically, we extracted total winter precipitation (cumulative precipitation during December, January, and February of the previous winter) and the spring (March, April, and May) mean maximum temperature (*T*_max_). Winter precipitation was selected because the California Floristic Province, where *S. tortuosus* occurs, receives the majority of annual rainfall during winter months. Maximum temperature was selected instead of mean or minimum temperatures because this parameter has been shown to have a higher predictive power (*R* ^2^) than other temperature parameters in large‐scale phenological models (Park and Mazer, 2018).

### Statistical analyses

In the analyses presented here, we analyzed a small proportion (*n* = 119 specimens) of all *S. tortuosus* specimens available from the CCH for which the exact collection date (day, month, and year) was recorded (Fig. 3). Despite this seemingly small sample size, Park and Mazer (2018) demonstrated that increasing the number of specimens included in pheno‐climatic models past 100 specimens does not further improve model predictive power. For each specimen, that date was converted into a day of the year of collection (DOY; e.g., July 4 is day 185, or 186 on leap years). The DOY was evaluated for normality with a quartile‐quartile plot.

We used multiple linear regressions to investigate the relationship between DOY and local climatic conditions in the year of collection using two distinct models. In the first model, we made no attempt to account for variation in phenological status among sheets; as such, we did not include PI in this model. This model represented the manner in which phenological responses to local climate have historically been examined using herbarium specimens. In the second model, however, we controlled for variation in phenological status among sheets by including the PI for each specimen as a main effect in the model. By comparing the results of this second model against the baseline model that does not incorporate PI as a main effect, we were able to evaluate the degree to which the addition of PI as a main effect improved model performance or adjusted the predicted phenological responsiveness to differences in local climate. We validated the predictive power of both models using 10‐fold cross‐validation. Multiple linear regression analyses were performed in JMP Pro 13 (SAS Institute, Cary, North Carolina, USA) and multiple regressions using 10‐fold cross‐validation were performed using Python version 2.7.11 (Oliphant, 2007).

## RESULTS

**Figure 4:**
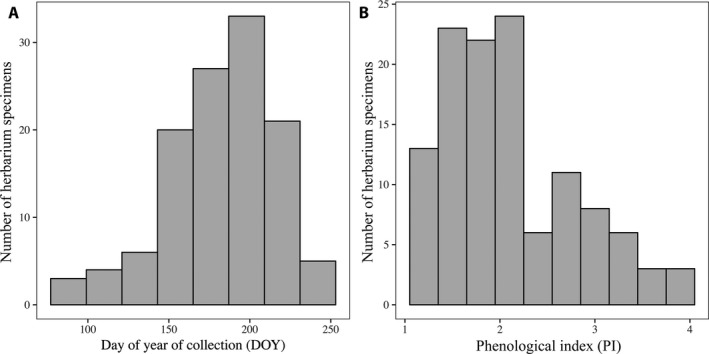
The distribution of (A) the day of year of collection and (B) the phenological index of the *Streptanthus tortuosus* herbarium specimens analyzed here (*n* = 119 specimens). The mean DOY is 182.03 days and the mean PI is 2.10.

The *S. tortuosus* herbarium specimens analyzed here were collected between 12 July 1898 and 9 May 1999. The DOY ranged from 88 to 253 (29 March to 10 September; $\overset{¯}{x}$ = 182.03 or 1 July, SD = 34.55 days; Fig. 4A). The PI ranged from 1.05 to 3.89 ($\overset{¯}{x}$ = 2.10, SD = 0.69; Fig. 4B). Despite a relatively small sample size (*n* = 119 specimens), we were able to capture a wide variety of collection dates and phenological progressions in our sample (Fig. 4). The mean number of plants per herbarium sheet used in this study was 2.67 (SD = 2.04 plants).

To investigate the relationship between DOY and climate, we ran two multiple linear regressions. The first model (Model 1) includes temperature and precipitation parameters as main effects, whereas the second model (Model 2) includes the same climatic parameters in addition to the PI as main effects. Model 2 explains 31% more variation in DOY than Model 1 (*R* ^2^ = 0.47 vs. 0.36, respectively) and has a lower overall corrected Akaike information criterion (AICc, 1111.18 vs. 1132.97; Table 2). In order to test the power of these models to predict the DOY of collection among specimens not used in model construction, both models were validated using 10‐fold cross‐validation. The resulting models resulted in an even more dramatic increase in predictive power among models that incorporated PI relative to those that did not (measured by the mean *R* ^2^ across all folds; Model 1 *R* ^2^ = 21%; Model 2 *R* ^2^ = 41%; Appendix S1).

**Table 2:** Results from the multiple regressions designed to detect the effects of *T*_max_ and precipitation on the DOY of specimen collection including (A) the effect tests and (B) the parameter estimates. Model 1 does not account for variation in phenological stage among specimen sheets while Model 2 accounts for this variation by including the phenological index (PI) as a main effect.

A.				
Analysis of variance source	df	Sequential SS	F ratio	*P* value
Model 1				
Winter precipitation	1	5389	7.05	<0.01
Spring *T*_max_	1	48,477	63.47	<0.01
Error	116	88,596		
*R* ^2^				0.36
AICc				1132.97
Model 2				
PI	1	16,164	25.66	<0.01
Winter precipitation	1	7263	11.53	<0.01
Spring *T*_max_	1	46,365	73.61	<0.01
Error	115	72,432		
*R* ^2^				0.47
AICc				1111.18

B.				
Parameter estimates term	Estimate	SE	*t* ratio	Prob > |*t*|
Model 1	** **	** **	** **	** **
Intercept	218.09	7.77	28.06	<0.01
Winter precipitation	0.017	0.01	2.66	<0.01
Spring *T*_max_	−4.23	0.53	−7.97	<0.01
Model 2				
Intercept	179.2	10.43	17.19	<0.01
PI	17.08	3.37	5.07	<0.01
Winter precipitation	0.02	0.01	3.4	<0.01
Spring *T*_max_	−4.14	0.48	−8.58	<0.01

Note

AICc = corrected Akaike information criterion; PI = phenological index; *T*_max_ = maximum temperature.

Both models detected a significant and quantitatively similar relationship between DOY and spring maximum temperature; DOY advances with increased temperature. Model 1 parameter estimates indicate that DOY advances 4.23 ± 0.53 days/°C (F_1,116_ = 63.47, *P* < 0.01), whereas Model 2 parameters indicate that flowering time advances 4.14 ± 0.48 days/°C (F_1,115_ = 73.61, *P* < 0.01; Table 2).

In both models, DOY is delayed in response to increased spring precipitation. Model 1 parameter estimates indicate that flowering time is delayed by one day for every 58.8‐mm increase in winter precipitation (0.017 ± 0.01 days/mm of precipitation, F_1,116_ = 7.05, *P* < 0.01), whereas Model 2 detected that DOY is delayed by one day for every 50‐mm increase in winter precipitation (0.02 ± 0.01 days/mm, F_1,115_ = 11.53, *P* = < 0.01; Table 2).

Model 2 indicates that PI increases with DOY, independent of variation in the climatic variables included in the model. Among the herbarium specimens, a specimen advances one phenological stage (e.g., from buds to flowers or from flowers to immature fruits) every 17.08 ± 3.37 days (F_1,115_ = 25.66, *P* < 0.01; Table 2B). This means that, on average, a mean of 51.24 days elapses between the appearance of buds and the complete conversion of these buds to mature fruits.

**Figure 5:**
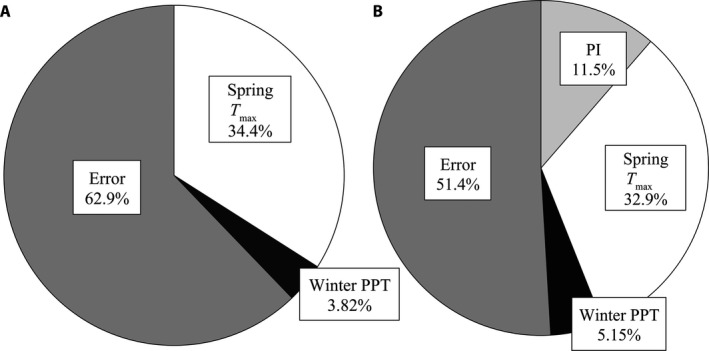
The proportions of variance attributed to each main effect and the model error in (A) Model 1 and (B) Model 2. Including the phenological index (PI) in the model reduces the proportion of total variance in day of year of collection attributed to error. PPT = precipitation; *T*_max_ = mean maximum temperature.

Although both models predicted qualitatively similar relationships between DOY and climate, the proportion of variance in DOY explained by each parameter in the models differed. In Model 1, the error variance in DOY was 22% higher than in Model 2 (62.9% vs. 51.4%, respectively; Fig. 5). Model 2 has a lower portion of unexplained variance because some of the unexplained variance in Model 1 is explained by the PI in Model 2. The PI explains 11.5% of the variance (Model 2; Fig. 5B). Spring maximum temperature explains a lower proportion of the total variance in Model 2 than in Model 1, likely because some of the variance in PI was incorrectly attributed to spring *T*_max_ in Model 1 (32.9% vs. 34.4%, respectively; Fig. 5).

Including the PI allows us to use the parameterized pheno‐climatic model to predict the day of year of peak flowering of *S. tortuosus* at a given location under either current conditions or future projected climate change scenarios. Given Model 2, for example, we may predict the day of year on which *S. tortuosus* will be at peak flower (estimated here by a value of PI = 2.5) at a given location with a given set of climatic parameters, in this case winter precipitation (winter PPT) and maximum spring temperature (spring *T*_max_), from the following equation:

(2) $$\text{DOY}=17.08^{*}\left(2.5\right)+0.02^{*}\left(\text{winter}\;\text{PPT}\right)-4.14^{*}\left(\text{spring}\;T_{\max}\right)+179.2$$

where 2.5 is a hypothetical value of PI for peak flowering. By inputting forecasted temperature and precipitation parameters for a given location from projected climate models, we can predict a species peak flowering time—or any other phenophase identified by a particular value of the PI—at that location.

## DISCUSSION

The work presented here was motivated by four primary objectives. First, we developed a protocol to score the phenological status of imaged herbarium specimens by first counting the number of reproductive organs representing different developmental stages (e.g., buds, open flowers, immature fruits, and mature fruits). This process was facilitated by the use of Cell Counter, a plugin available through the free image processing and analysis software ImageJ. This protocol provides users a fast and easy way to reliably score imaged herbarium specimens. Second, we used these counts to develop a new quantitative metric of a specimen's phenological status: the phenological index. We then demonstrated how it can be used to construct and improve pheno‐climatic models in our analysis of a herbarium‐derived data set composed of *S. tortuosus* specimens. We found that while including the PI as an independent variable in a pheno‐climatic model does not appear to dramatically alter the resulting model coefficients, it does provide a substantial improvement to the model's predictive power by accounting for variation in DOY caused by collection of specimens at different phenological stages. Third, we tested a series of predictions concerning the phenological response of *S. tortuosus* to local climate. We found that warmer spring maximum temperatures and drier winters during the year of specimen collection advance the reproductive phenology of *S. tortuosus* across its range. Finally, we demonstrated how pheno‐climatic models constructed with the PI as an independent variable can be used to estimate the length of the reproductive period as well as forecast the day of year of onset of any reproductive phase for any given set of climatic conditions.

### Relationship between climate and flowering date

Even given the relatively small sample size analyzed here, we were able to detect highly significant associations between local climatic conditions in the year of specimen collection and the DOY of our focal specimens of *S. tortuosus*. The DOY of sampled herbarium specimens advances with increased temperature and is delayed with increased precipitation, which corroborates our predictions concerning the relationship between flowering date and climate. The sensitivity of DOY to temperature observed in *S. tortuosus* is consistent with that observed in other herbarium‐based studies of intraspecific variation in phenology in relation to climate. For example, Matthews and Mazer (2016) found that, among herbarium specimens collected in flower, the sensitivity of *Trillium* *ovatum* Pursh to temperature is −4.74 days/°C. Similarly, Gaira et al. (2014) investigated species’ sensitivity to temperature in *Rhododendron arboreum* Sm. using herbarium specimens and found that increasing temperature advanced flowering date (−4.26 days/°C). Both of these studies detected similar sensitivities to temperature to that detected in *S. tortuosus* (−4.14 days/°C in Model 2; Table 2B).

In many species, the relationship between phenology and precipitation remains unclear and can be highly species‐ or community‐specific (Hart et al., 2014; Munson and Sher, 2015; Rawal et al., 2015; Matthews and Mazer, 2016; Hufft et al., 2018). Similar to the pattern detected here, Matthews and Mazer (2016) found that increased precipitation delays flowering time in *T. ovatum*. Across a diverse group of alpine species, Hufft et al. (2018) also found that precipitation delayed flowering time (0.02 days/mm). Surprisingly few herbarium‐based studies have investigated the impact of precipitation on phenology. Moreover, none have investigated this relationship within water‐limited ecosystems such as California, where precipitation may be expected to be an important factor affecting reproductive phenology (Mazer et al., 2015). Expanding herbarium‐based studies to investigate phenology–precipitation relationships will help us to gain a deeper understanding of how species will be impacted by future climate changes. Newly available high‐resolution climate data (e.g., PRISM and ClimateNA [https://sites.ualberta.ca/~ahamann/data/climatena.html]) will facilitate the testing of more complex models and the detection of more subtle relationships between climate and the timing of distinct phenophases (Wang et al., 2016).

### Calculating and incorporating the phenological index into phenological models

Here, we provide a simple and readily available method to score imaged herbarium specimens using the free image analysis software program, ImageJ, and the available plugin, Cell Counter. Cell Counter allows its user to use point‐and‐click movements to accurately count the numbers of reproductive organs representing each of any number of distinct phenological phases, as specified by the user. Because of the ease and simplicity of this protocol, it could be easily incorporated into workflows that include scoring by citizen scientists, especially with the forthcoming widespread availability of imaged herbarium specimens through data aggregators such as Integrated Digitized Biocollections (iDigBio; http://www.idigbio.org) and Global Biodiversity Information Facility (GBIF; http://www.gbif.org).

The counts derived from Cell Counter may then be used to calculate a PI that represents a weighted mean of the combined counts (as demonstrated in Equation 1). This protocol can be adapted to many species and would work especially well for those with clear, large, and easily counted reproductive structures or compound reproductive structures (such as those found in the Asteraceae family). Species that may be difficult to score are those with small or indistinct reproductive structures or those for which the transitions between phenophases are ambiguous.

When PI was included in the pheno‐climatic model tested here (Model 2), this variable accounted for 11.5% of the variance in flowering date among *S. tortuosus* specimens (Fig. 5B). A far higher proportion of the total variance in DOY (38.05%) was explained by climatic parameters. Inclusion of the PI reduced the overall error in the model while improving its predictive power. However, in the data set analyzed here, including the PI did not drastically change the regression coefficients of the climatic parameters in the model. Similarly, Pearson (2019) and Ellwood et al. (2019) both found that models including finer‐scale phenological coding (e.g., including only those specimens with >50% flowers) were statistically similar to those models that did not include this finer‐scale coding. Thus, these results indicate that herbarium‐based phenological models that do not incorporate PI still provide accurate assessments of phenological responsiveness to local climate. At the same time, the inclusion of PI not only reduces the amount of unexplained variance in the resulting pheno‐climatic model, but also increases the power of such models to predict the timing of specific phenological events such as flowering onset, peak flowering, or flowering termination. Additionally, inclusion of the PI allowed us to estimate the average total length of the reproductive phase of *S. tortuosus* specimens as ~51.24 days long. This estimate offers a way to test predictions concerning how climate may influence not only the mean flowering date of focal species but also the length of the reproductive phase, which could be especially useful for investigating intraspecific geographic, temporal, and/or climate‐induced variation in the duration of the reproductive phase. For example, we may predict that, among widespread montane species such as *S. tortuosus*, specimens collected from more alpine environments will have a shorter reproductive phase than those collected from lower elevations due to the shorter growing season at high elevations (Hunsaker et al., 2012). This prediction could be tested by separating conspecific data sets into groups of specimens representing differing elevations (e.g., high and low elevation). The regression coefficient of the PI may differ between models constructed using these data sets, thereby demonstrating how the duration of reproduction may also differ among them.

Including the PI in pheno‐climatic models allows us to create a predictive model that we may use to forecast the day of year of peak flower (or for plants representing any specific value of the PI) for *S. tortuosus*. By including only two climatic parameters, we were able to construct a model that predicts the day of year of peak flowering among our sampled herbarium specimens with 47% accuracy (Table 2B, Equation 2). With a larger data set, we can improve these models by including other climatic parameters such as relative humidity, vapor‐pressure deficit, or winter or summer temperature. Given the millions of herbarium specimens now available for research, these pheno‐climatic models can be constructed for many species, and ultimately combined to give us a broader understanding of how climate change may affect not only the reproductive phenology of individual species but also the collective phenology of plant communities (Park and Mazer, 2019).

One of the main goals of herbarium‐based studies is to investigate long‐term shifts in flowering date through time to determine whether the seasonal cycles of plants have been affected by recent temperature increases. Some of these studies have successfully detected advances in flowering date through time (Molnár et al., 2012; Panchen et al., 2012; Searcy, 2012), whereas others have failed to find an effect even for species that were found to be sensitive to changes in temperature (Hart et al., 2014; Davis et al., 2015; Park and Schwartz, 2015). For example, Hart et al. (2014) found that the flowering date of *Rhododendron* species was sensitive to changes in annual average temperature (−2.27 days/°C); therefore, they expected that because mean temperature had increased during the study period (1952–2009), they would detect a temporal advance in flowering date. However, they failed to detect a statistically significant phenological shift. The variation in phenological stage among specimens may have obscured the true relationship across the sampled decades. Including the PI may be especially useful in models designed to detect shifts in flowering date through time because such shifts are likely to be small and difficult to detect. Consequently, reducing the error variance in the model due to variation among specimens in their phenological status will likely improve our ability to detect temporal shifts in flowering date while also helping to improve the fit and accuracy of pheno‐climatic models.

Because of their extensive geographic, temporal, and taxonomic record of plant occurrences, herbarium specimen–based studies provide a promising way to investigate the relationship between flowering time and climate. The new metric introduced here, the phenological index, should reliably reduce error variance in flowering date derived from herbarium collections and improve the predictive capacity of phenological models. Although scoring reproductive phenology using the ImageJ protocol described here does require considerable effort, promising improvements in the automated annotation of specimens with deep learning will expedite the scoring process and ultimately provide us with high‐resolution phenological data with which to construct phenological indices (PIs) and to improve pheno‐climatic models (Lorieul et al., 2019).

In our multivariate models for *S. tortuosus*, the inclusion of PI as an independent variable reduced the resulting error variance in the DOY among specimens while increasing the model's predictive power and decreasing the AICc. The PI also provides a way to quantify the reproductive period of plants from herbarium specimens and allows us to estimate not only how climate affects flowering dates but also how climate may affect the length of the reproductive period. Moreover, pheno‐climatic models constructed with the PI can be used to forecast the day of year of a specific phenophase, given any specified set of climatic parameters.

## Supporting information


**APPENDIX S1.** Ten‐fold cross‐validation for pheno‐climatic Models 1 and 2.

## Figures and Tables

**Figure A1:**
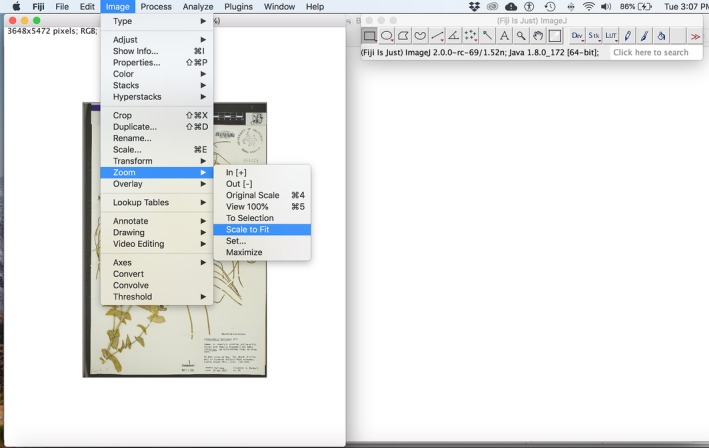
To increase the size of the image in the program, select “Image **→** Zoom **→** Scale to fit” from the menu.

**Figure A2:**
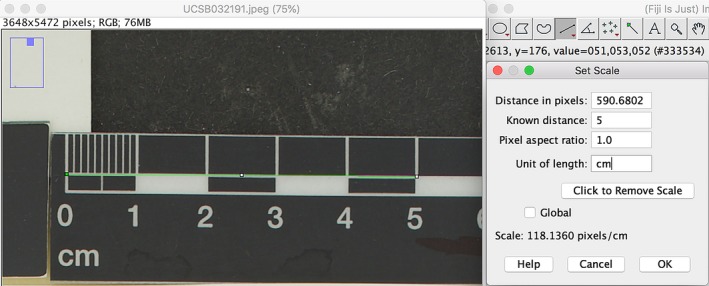
Use the straight segment tool to draw a line along the ruler in the imaged herbarium specimen (shown here as a green line from 0 to 5 cm). Fill out the dialog box with the known length and units of the segment you drew.

**Figure A3:**
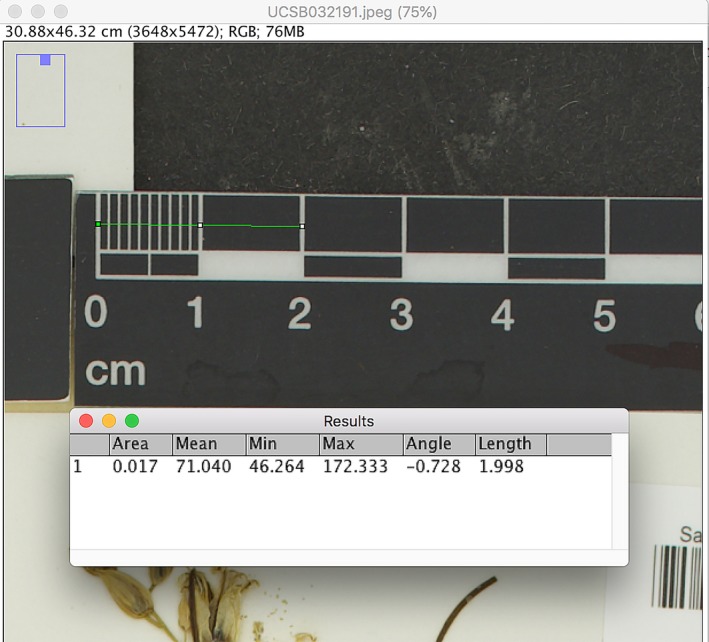
Test the measurement calibration after you set the scale using the ruler on the imaged herbarium specimen. Here, a 2‐cm length is drawn along the scale bar in green. You can see the measured length in the “Length” column. The units are centimeters, which were chosen during Part III, Step 1 above.

**Figure A4:**
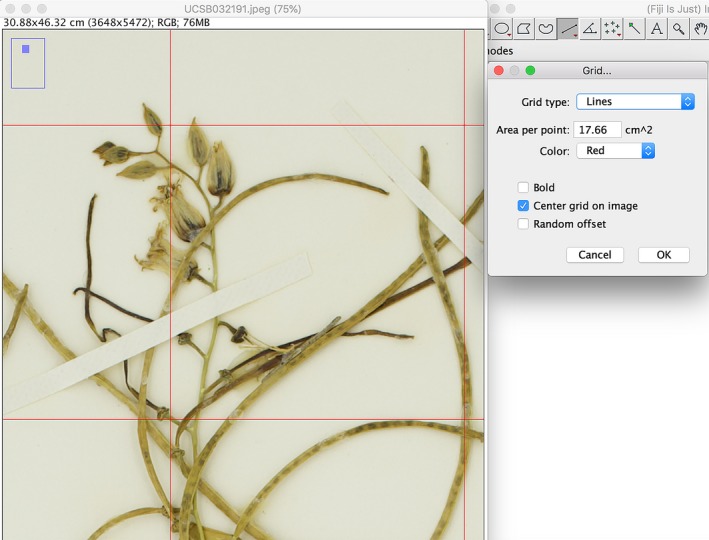
To keep track of regions within the imaged herbarium specimen, add a grid by selecting “Analyze **→** Tools **→** Grid” from the menu.

**Figure A5:**
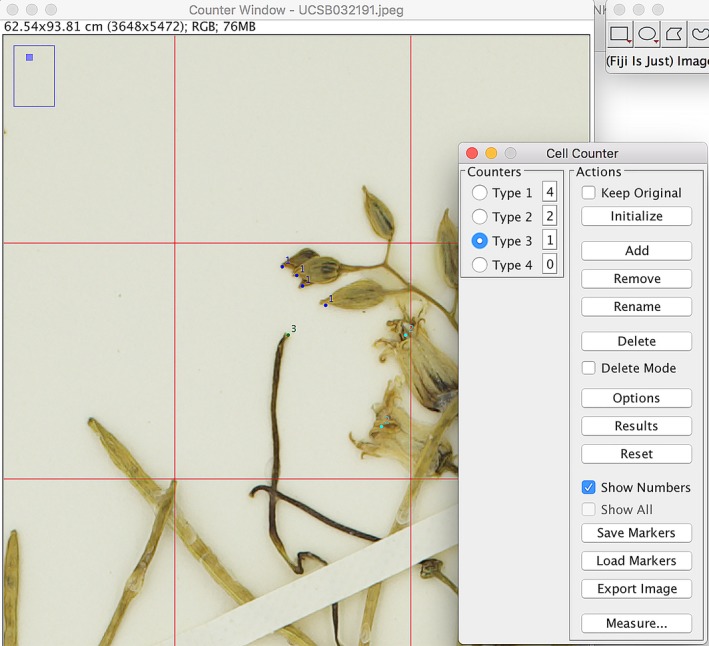
All reproductive organs are scored in the center grid. The number of reproductive organs of each type is shown as in the Cell Counter window.

**Figure A6:**
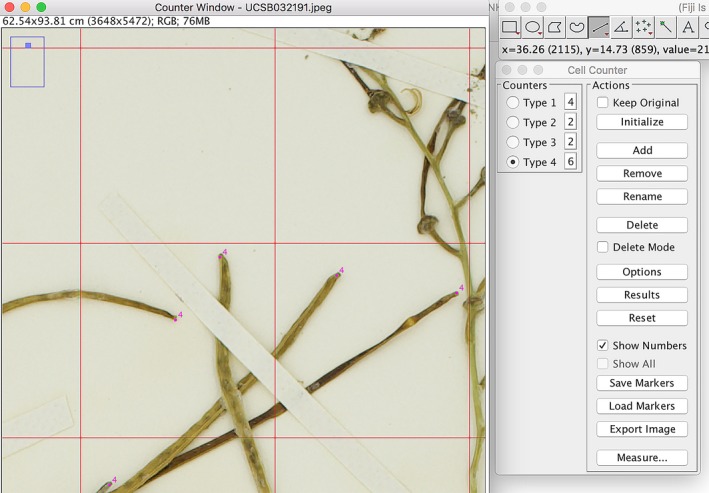
The markers are placed at the distal ends of mature and immature fruits.

**Figure A7:**
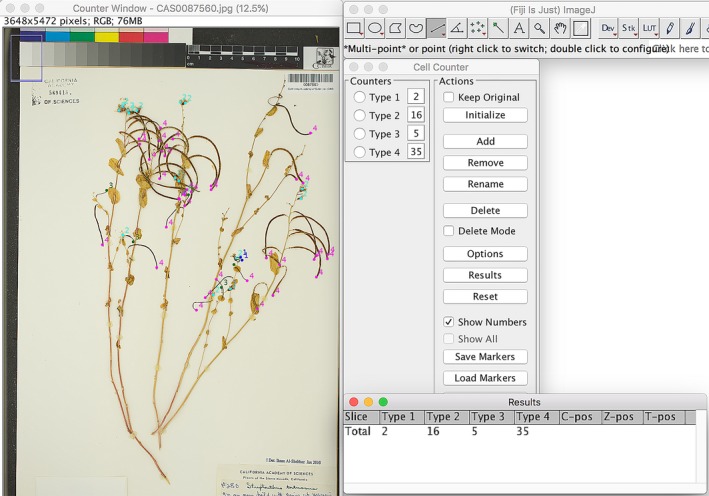
A scored specimen. Click the “Results” button on the Cell Counter dialog box to see the number of reproductive organs scored in each phenophae.

**Figure A8:**
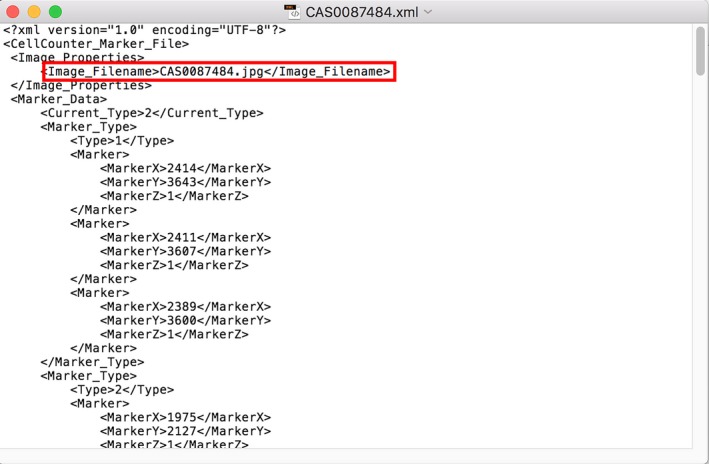
In the XML file, the “Image_Filename” (shown in the red box) should match exactly the name of the imaged herbarium specimen to which the scored points belong.

## Data Availability

All data associated with this manuscript are accessible on Zenodo (Love et al., 2019).

## APPENDIX 1 Scoring phenology on *Streptanthus tortuosus* herbarium specimen images using the ImageJ plugin Cell Counter.

**Required software:** Download ImageJ at https://imagej.nih.gov/ij/download.html. Choose the operating system appropriate for your computer and follow the installation instructions.

OR Download Fiji (comes with Cell Counter already installed): https://imagej.net/Fiji/Downloads

Download the required Cell Counter plugin here: https://imagej.nih.gov/ij/plugins/cell-counter.html

**Part I: Installing the software and integrating the plugin (on Mac)**

1. Download ImageJ and drag the entire downloaded folder to your “Applications” folder. (Note: If you downloaded Fiji, you can skip the plugin integration steps here because Cell Counter is already installed. If this is the case, skip to step 4.)
2. Open the “ImageJ” folder in “Applications.” Open the “Plugins” folder and then open the “Analyze” folder. Drag the “Cell_Counter.jar” file plugin that you downloaded from your downloads folder into the “Analyze” folder.
3. There is an apparent bug in the software that prevents the immediate use of the plugin. To use the plugin, first drag the ImageJ application (the application itself has a microscope icon) out of the “ImageJ” folder in which it is located. For example, drag it into the “Applications” folder and then drag it back to the “ImageJ” folder.
4. Launch the plugin by accessing the Plugins menu: Open ImageJ **Plugins → Analyze,** where you should see the **Cell Counter** plugin as an option.

**Part II: Opening and navigating images**

1. Open ImageJ.
2. Assemble your herbarium specimen images (saved as JPEG files) in a conveniently located folder. Open a specimen image in ImageJ either by using the File menu (Select “**File → Open**” and navigate to the folder with the images to select the desired image) or by dragging an image file onto the ImageJ icon.
3. Adjust the scale of the image so you can see it large and clear on your screen. Do this by clicking the lower left corner of the image and enlarging the viewing window to about half of your screen (be careful not to cover the tool bar completely). You will notice that the image does not scale with the window. To fix this, select “**Image → Zoom → Scale to fit**” (Fig. A1).
4. Practice navigating around the image using the tool bar. Select the “magnifying glass” tool and practice zooming in and out using the + and – keys. Select the “pan” tool (hand symbol) to practice moving around within the image viewer. You can also use the pan tool by holding down the spacebar and moving your mouse.

**Part III: Setting the scale**

1. Set the scale so you can overlay a digital grid on the specimen image. Zoom into the black and white scale bar at the top of the specimen's image. Select the “Straight” segment tool from the tool bar and use the cursor to drag a line along the scale from 0 to 5 cm. Once this line is created, you may set the scale by selecting (from the Menu bar) **Analyze → Set Scale.** In the dialog box, fill in “Known distance” with the value “5” and “Unit of length” with “cm”. Click the “Global” box to set this measurement for your entire session. Then click “OK” (Fig. A2).
2. ***Test your scale.*** Using the “Straight” segment tool, draw a line along the scale bar. In the example in Fig. A3, a green line has been drawn along a 2‐cm length of the scale bar. Then select **Analyze → Measure**. A “Results” box will be displayed. Check to make sure that the measurement shown in the “Length” column matches the scale measurement (Fig. A3). The units in the Length column are the units you set in the “Set Scale” dialog box during Part III, step 1.

**Part IV: Scoring images**

1. Add a grid to the image to help you navigate and keep track of regions within the specimen image that you have already counted by selecting **Analyze → Tools → Grid**. The “Grid” dialog box shown in Fig. A4 generated a grid comprising17.58‐cm^2^ squares, but the size of the grid cells may be adjusted depending on the user's preference. **Note:** If an error message appears when adding the grid, then you need to set the scale first (see Part III).
2. Now, you are ready to start counting the numbers of each category of reproductive organ. If there is more than one plant mounted on a herbarium sheet, then count the reproductive organs on each plant separately. Steps 3–10 provide instructions for how to conduct the counts for the first plant; instructions for how to count the reproductive organs on the subsequent plants are presented at Step 12. You will use the “Cell Counter” plugin to keep track of your counts. Select **Plugins → Analyze → Cell Counter.** This will display the Cell Counter dialog box.
3. In the dialog box, check the “Keep Original” and “Show Numbers” boxes and then press “Initialize” to start the counting. This will create and open a copy of the image called “Counter Window – *file name*”.

**Note:** You cannot use the measure tool while you are using Cell Counter. If you need to measure a bud to see if it is greater than 2 mm (or whichever threshold size you are using to identify a given organ type or phenophase), you can measure it on the original image. To measure a bud on the *original* image, select the Straight segment tool and draw a line along the length of the bud. Select **Analyze → Measure.** This will display a results table with the length of the line in the last column. Recall that, in this example, the units are in centimeters (i.e., a length of 0.68 = 6.8 mm). Any bud greater than 0.2 cm in length should be counted. You do not need to save measurements.

1. You will have to expand the window and select **Image → Zoom → Scale to fit** to make the new Counter Window image as large as possible.
2. You can begin counting in the first grid cell that contains reproductive organs (Fig. A5). Select “Type 1” to start counting the buds. Click on a bud to add a marker. For consistency, always add the marker to the tip of the bud. For each marker you add, notice that the number in the Cell Counter window to the right of “Type 1” increases by one. Buds will be classified as “Type 1”, open flowers as “Type 2”, immature fruits as “Type 3”, and mature fruits as “Type 4.” Because we are only counting four types of reproductive organs, you can delete the other marker types by clicking “Remove” in the Cell Counter window.

**Note:** If you place an erroneous marker, you can press the “Delete” button, which will delete the last point you made, or you can check the “Delete Mode” box, which will allow you to delete any point of the selected marker on which you click using the cursor.

1. After you count the buds in the first grid cell, change the counter to “Type 2” to count the flowers. Add a point to the top each flower.
2. Change the counter to “Type 3” to count the immature fruits. In each grid cell, place a marker at the distal end of each immature fruit (Fig. A6). **Note:** Because *Streptanthus* fruits often span more than one grid cell, it is important to make consistent decisions regarding in which grid cell a given fruit is counted. Given that leaves or other foliage often obscure the bases of fruits, identifying (and counting) each fruit by placing a marker at its distal tip can help users to avoid missing a fruit or counting a given fruit twice. In the image below, Cell Counter has been used to place a bright magenta marker at the distal end of each mature fruit in the grid cell displayed.
3. Now add a marker at the distal end of the mature fruits in the grid cell. For mature fruits, change the counter to Type 4.
4. Progress to the next grid cell and change the counter back to Type 1 and add a point to all of the buds. Then continue adding markers to count the open flowers, immature fruits, and mature fruits in this cell, remembering to change the “Type” as needed for each phenophase. You should follow the grids from top to bottom, left to right for each plant. **Tip:** Remember that you may “pan” around the image by holding down the spacebar and dragging the image. You can use the scroll bar to scroll up and down through the image. You can zoom in and out by using the command key ⌘ and the +and ‐ keys.
5. When you are finished adding markers to all of the reproductive organs borne by the first plant examined on the herbarium sheet, click the “**Results**” button in the Cell Counter dialog box (Fig. A7). This will open a new window in which the numbers of each phenophase are displayed. Copy and paste these numbers into a spreadsheet.
6. Save the location of the count markers by clicking the “Save Markers” button in the “Cell Counter” dialog box. This will save the counts as an XML file. Save the count file as “AccessionNumber_plant1”. Note that the counts for the remaining plants on this herbarium sheet should be saved as “AccessionNumber_plant2”, “AccessionNumber_plant3”, etc. Be sure to delete the “Counter Window” text in the file name.
7. After saving the counts recorded from the first plant, you may move on to the remaining plants on the herbarium sheet (if present). In the Cell Counter dialog box, click “reset” to delete the points and to reset the counters. Start the protocol again at Part IV, Step 5. Do NOT click initialize again. Continue for each plant on the sheet, saving the points recorded on each plant as an individual XML file.
8. When you are finished counting a single sheet, move the XML count files to a folder dedicated to these XML files.

**Part V: Recalling and editing XML files**

1. To recall the XML marker files for an image, first open the JPEG of the herbarium specimen sheet in ImageJ or Fiji.
2. Go to **Plugins → Analyze → Cell Counter** to open up the Cell Counter dialog box.
3. Click “Initialize” in the Cell Counter window.
4. Click “Load Markers” and navigate to the folder where the XML files are saved and select the corresponding XML file for the current image.

**Note:** If you receive the error “These Markers do not belong to the current image” then there may be a fixable error in your XML file. To fix the error, open the XML file in any text editor program (e.g., Notepad, TextEdit, or Fiji) and inspect the Image_Filename listed in the XML file (Fig A8). The Image_Filename should match *exactly* the file name of the herbarium specimen image. Any extra text such as “Counter Window” or “(1)” will cause an error. Extra text may appear in the Image_Filename if, for example, the user clicks the “Initialize” button twice while scoring an image. Extra text may also appear in the Image_Filename if the image has been downloaded more than once to a given folder (indicating a new version number). You may modify or modify the image file name that appears in the XML file to make sure that it matches exactly the file name of the corresponding saved image. In the example in Fig. A8, loading XML markers to an image with the name “CAS0087812.jpg” will be successful because the Image_Filename (“CAS0087812.jpg” in the red box) matches our image file name exactly.

1. You can also edit the XML files. To do this, recall the XML files to the image as described above. You can add new markers or delete current markers using the “Delete Mode” function. Click “Save Markers” and save a new version of the XML.
