# Supplementary material for: A new phenological metric for use in pheno‐climatic models: A case study using herbarium specimens of Streptanthus tortuosus
Source: Appl Plant Sci. 2019 Jul 12;7(7):e11276. doi: 10.1002/aps3.11276 (PMC6636619; doi:10.1002/aps3.11276)
Supplement: Supplementary file 1 — APPENDIX S1. Ten‐fold cross‐validation for pheno‐climatic Models 1 and 2. [file APS3-7-e11276-s001.pdf]

DOI

APPENDIX S1. Ten-fold cross-validation for pheno-climatic Models 1 and 2.

In [69]:
```
import pandas as pd
import numpy as np
from sklearn import linear_model
from sklearn.model_selection import KFold
```

In [94]:
```
def KFoldScore(X, y, splits, seed = None):
    ''' Conduct k-fold cross validation on dataset using linear regres
sion,
    returns the R2 values of each iteration (alongside mean R2 value a
cross all iterations),
    the coefficients of each model iteration,
    and the intercepts of each model iteration

    #Param X: Dataframe containing all explanatory variables to be use
d in model
    #Param y: Series of response variable
    #Param splits: number of splits to be used in cross-validation
    #Param seed: seed to be used in randomization, if desired
    '''


    X = np.asarray(X)
    y = np.asarray(y)

    # create training and testing vars
    kf = KFold(n_splits=splits, random_state=seed, shuffle=False)
    kf.get_n_splits(X)

    R2List = []
    coeffsList = []
    interceptList = []


    print "Iterated R2 values: "

    #iterate model across all folds
    for train_index, test_index in kf.split(X):
        #print("TRAIN:", train_index, "TEST:", test_index)

        X_train, X_test = X[train_index], X[test_index]
        y_train, y_test = y[train_index], y[test_index]
        lm = linear_model.LinearRegression()
        model = lm.fit(X_train, y_train)
        R2_Out = model.score(X_test, y_test)
        R2List.append(R2_Out)
        coeffsList.append(model.coef_)
        interceptList.append(model.intercept_)
        print R2_Out
        meanR2 = sum(R2List) / float(len(R2List))
    print "\nmean R2 = ", meanR2

    return R2_Out, coeffsList, interceptList
```

In [95]:
```
#Read in Dataset
in_Data = pd.read_csv('Streptanthu tortuosus Herbarium Data.csv')
in_Data.head()
```

Out[95]:

	Barcode	DOY	Latitude	Longitude	PI	Elevation (m)	Winter PPT YOC	Spring Tmax YOC
0	65004	124.0	36.762222	-119.211389	1.980523	580	222.93	10.950000
1	87514	104.0	39.590800	-121.548300	1.745098	358	812.26	19.900000
2	87515	174.0	39.681944	-121.346389	2.409180	975	1529.34	17.266667
3	87549	99.0	38.789400	-120.859200	1.285714	379	636.67	20.233333
4	87550	179.5	38.884960	-120.092318	2.621176	2229	477.94	9.966667

In [96]:
```
#extract Pandas Dataframe of climate & PI
PI_Data = pd.DataFrame(in_Data, columns=['PI','Winter PPT YOC', 'Sprin
g Tmax YOC'])
PI_Data.head()
```

Out[96]:

	PI	Winter PPT YOC	Spring Tmax YOC
0	1.980523	222.93	10.950000
1	1.745098	812.26	19.900000
2	2.409180	1529.34	17.266667
3	1.285714	636.67	20.233333
4	2.621176	477.94	9.966667

In [97]:
```
#extract Pandas Dataframe of climate Data Only, Excluding PI
NoPI_Data = pd.DataFrame(in_Data, columns=['Winter PPT YOC', 'Spring T
max YOC'])
NoPI_Data.head()
```

Out[97]:

	Winter PPT YOC	Spring Tmax YOC
0	222.93	10.950000
1	812.26	19.900000
2	1529.34	17.266667
3	636.67	20.233333
4	477.94	9.966667

DOI

In [98]:
```
#Extract Series of DOYs
y = in_Data.DOY
y.head()
```

Out[98]:
```
0    124.0
1    104.0
2    174.0
3     99.0
4    179.5
Name: DOY, dtype: float64
```

In [102]:
```
#Conduct 10-Fold cross validation on model including PI, Winter PPT YO
C, and Spring Max YOC
R2, Coeffs, Intercepts = KFoldScore(PI_Data = df, y = y, splits = 10,
seed = 12345)
```

```
Iterated R2 values:
0.658766914481
0.607945317349
0.146732978056
-0.0530450253563
0.239234449229
0.593785620593
0.662896538165
0.448800298687
0.206169803148
0.576136710328

mean R2 =  0.408742360468
```

In [103]:
```
#Conduct 10-Fold cross validation on model including PI, Winter PPT YO
C, and Spring Max YOC
R2, Coeffs, Intercepts = KFoldScore(X = NoPI_Data, y = y, splits = 10,
seed = 12345)
```

```
Iterated R2 values:
0.562778849909
0.331424462396
-0.0638936899472
-0.348753092625
0.444302052112
0.360790932413
0.460198712373
0.0576172713816
-0.111226264244
0.412521650428

mean R2 =  0.21057608842
```
